# Biosystems Study of the Molecular Networks Underlying Hippocampal Aging Progression and Anti-aging Treatment in Mice

**DOI:** 10.3389/fnagi.2017.00393

**Published:** 2017-12-06

**Authors:** Jiao Wang, Qian Li, Yanyan Kong, Fangfang Zhou, Jie Li, Weihao Li, Kai Wang, Ting Wu, Yihui Guan, Jiang Xie, Tieqiao Wen

**Affiliations:** ^1^Laboratory of Molecular Neural Biology, School of Life Sciences, Shanghai University, Shanghai, China; ^2^Position Emission Computed Tomography Center, Huashan Hospital, Fudan University, Shanghai, China; ^3^Shanghai Key Laboratory of Molecular Andrology, Institute of Biochemistry and Cell Biology, Shanghai Institutes for Biological Sciences, Chinese Academy of Sciences, Shanghai, China; ^4^Shanghai Stem Cell Group, Shanghai, China; ^5^School of Computer Engineering and Science, Shanghai University, Shanghai, China

**Keywords:** aging, immune response, hippocampal development, umbilical cord blood, molecular interaction network

## Abstract

Aging progression is a process that an individual encounters as they become older, and usually results from a series of normal physiological changes over time. The hippocampus, which contributes to the loss of spatial and episodic memory and learning in older people, is closely related to the detrimental effects of aging at the morphological and molecular levels. However, age-related genetic changes in hippocampal molecular mechanisms are not yet well-established. To provide additional insight into the aging process, differentially-expressed genes of 3- versus 24- and 29-month old mice were re-analyzed. The results revealed that a large number of immune and inflammatory response-related genes were up-regulated in the aged hippocampus, and membrane receptor-associated genes were down-regulated. The down-regulation of transmembrane receptors may indicate the weaker perception of environmental exposure in older people, since many transmembrane proteins participate in signal transduction. In addition, molecular interaction analysis of the up-regulated immune genes indicated that the hub gene, *Ywhae*, may play essential roles in immune and inflammatory responses during aging progression, as well as during hippocampal development. Our biological experiments confirmed the conserved roles of *Ywhae* and its partners between human and mouse. Furthermore, comparison of microarray data between advanced-age mice treated with human umbilical cord blood plasma protein and the phosphate-buffered saline control showed that the genes that contribute to the revitalization of advanced-age mice are different from the genes induced by aging. These results implied that the revitalization of advanced-age mice is not a simple reverse process of normal aging progression. Our data assigned novel roles of genes during aging progression and provided further theoretic evidence for future studies exploring the underlying mechanisms of aging and anti-aging-related disease therapy.

## Introduction

Aging is the process of an organism, particularly mammals, becoming older and characterized by a progressive loss of physiological integrity (Lopez-Otin et al., 2013). It is the result of a series of normal changes in gene regulatory networks over time, and is usually reflected by physiological changes (Lopez-Otin et al., 2013). The hippocampus aging, which contributes to the loss of spatial and episodic memory and learning in aging people, is closely related to the detrimental effects of aging at the morphological and molecular levels (Castellano et al., 2017).

The aged hippocampus may impair neural and cognitive function and lead to many neurological disorders such as Alzheimer’s disease (Stilling et al., 2014; Castellano et al., 2017), aphasia, agnosia, and Parkinson’s disease (Ghika, 2008). The aging hippocampus seems to suffer a generalized loss of synapses (Miller and O’Callaghan, 2003), which influence spatial memory and plasticity functions in multiple species (Moser and Moser, 1998; Hampson et al., 1999; Burke and Barnes, 2006). Moreover, a decrease in neuronal density (Mani et al., 1986; Keuker et al., 2003) and hippocampal volume (Driscoll et al., 2003) were found in the aging hippocampus, and these age-related losses occur in some degenerative conditions (West et al., 1994). Certain genes were predicted, for instance, a deubiquitinating enzyme- UCHL5 hub protein (Kikuchi et al., 2013), and neuropeptide arginine-vasopressin _(4-8)_ (AVP_4-8_) (Xiong et al., 2000) may be potential candidates for treating age-related disease. The increasing elderly population has increased labor costs and financial burden for both families and governments, thus, delaying or even partially reversing the aging process is of great interest (Castellano et al., 2017). However, due to the limited knowledge regarding aging, there are still numerous difficulties with different treatments for degenerative diseases linked to aging.

The most common theories for aging include telomere attrition, endocrine disorders, and oxidative stress (Patel et al., 2015). It has been suggested that low-grade inflammation, which is characterized by increased levels of inflammatory cytokines in response to environmental signals, contributes to aging processes (Nikodemova et al., 2016). The chronic inflammation eventually initiates immune-senescence in both the immune and central nervous system, resulting in the functional decline of the immune system with age (Regulski, 2017). Recently, advances in knowledge regarding neuroinflammation and immunity to brain aging have been reviewed (Di Benedetto et al., 2017). Since a plethora of research has been conducted in the exploration of aging mechanisms, recent research has been dedicated to elucidating ways in which to cease aging (Loffredo et al., 2013; Katsimpardi et al., 2014; Sinha et al., 2014; Castellano et al., 2017). GDF11 has been identified as a circulating factor in young mice that declines with age, and subsequently, GDF11 has been shown to be a rejuvenating factor for skeletal muscle (Sinha et al., 2014) and the brain (Katsimpardi et al., 2014). Furthermore, recent research has shown that tissue inhibitor of metalloproteinases 2, an umbilical cord plasma protein, provides a reservoir for the neuroplasticity-promoting process (Di Benedetto et al., 2017). Despite these research efforts, the molecular mechanisms underlying the progression of biological aging have not yet been established, likely due to the complexity of chronic aging progressions.

In the present paper, we aim to explore the molecular interaction mechanisms underlying aging using a biological systems approach that integrates computational and experimental methods. We assume that DEGs between advanced-age and young mice likely contribute to aging progression via certain mechanisms. Investigation into the molecular interactions of the genes identified potential aging regulators (or hub genes of the network). The key candidate genes inferred from network analysis were validated using qPCR. Our present study provides a more comprehensive analysis of the mechanisms underlying the aging process, and has laid the foundation for the exploration of genetic changes involved in the promotion of rejuvenation from advanced-age to young individuals.

## Materials and Methods

### Mice

Specific pathogen free C57BL/6 wild-type mice were fed under a controlled temperature (23°C) and kept on a 12 h light/dark cycle with food and water provided *ad libitum*. 3–4 mice were kept in individually ventilated cages. Littermate male mice (4 and 20 months old) were used, and three different tissue samples for RNA extraction were obtained per strain. All animal handling protocols were approved by the Animal Ethics Committee of Shanghai University.

### RNA Extraction and RT-PCR

Hippocampus was rapidly dissected in 0°C PBS and placed in RNA extraction solution (Promega, United States). RNA extraction was carried out in accordance with the manufacturer’s protocol. 2 μg total RNA and 4 μl 5X RT Master Mix (TaKaRa, Japan) were added to RNA-free water to a final reaction volume of 20 μl for cDNA synthesis under different temperature of 25°C for 5 min, 37°C for 30 min, 85°C for 10 s, and 12°C for 10 min. The target genes were quantified by real-time PCR using qPCR SYBR Green Master Mix (Yeasen, China). Each reaction contained 1 μl cDNA sample (100–200 ng/μl), 10 μl qPCR SYBR Green Master Mix, 0.8 μl (10 μM) designated primers, and RNA-free water to a final volume of 20 μl. The PCR conditions were as follows: 95°C for 30 s, 55°C for 20 s, 72°C for 30 s, 40 thermal cycles. The mRNA expression was normalized to GAPDH. The primers of each gene were shown in **Table 1**.

**Table 1:** Primers used in qPCR.

Gene name		Primer sequence (5′–3′)
YWHAE	Forward	GATTCGGGAATATCGGCAAATGG
	Reverse	GCTGGAATGAGGTGTTTGTCC
EZH2	Forward	AATCAGAGTACATGCGACTGAGA
	Reverse	GCTGTATCCTTCGCTGTTTCC
RARA	Forward	AAGCCCGAGTGCTCTGAGA
	Reverse	TTCGTAGTGTATTTGCCCAGC
HDAC1	Forward	AGTCTGTTACTACTACGACGGG
	Reverse	TGAGCAGCAAATTGTGAGTCAT
DCLK2	Forward	CCAAGAAGGCGCGGTTCTA
	Reverse	TGGGGCAGGTTCACATTGTC
TGM1	Forward	GCAATGAGATCTACATCCTCTTC
	Reverse	GAAGAGGATGTAGATCTCATTGC
CD74	Forward	TCACCTCCCAGAACCTGCA
	Reverse	TGCATCACATGGTCCT
VIM	Forward	GAGAAATTGCAGGAGGAGATGC
	Reverse	CAAGGTCAAGACGTGCCAGAGA
RBM3	Forward	CTTCGTAGGAGGGCTCAACTT
	Reverse	CAACAACCACCTCAGAGATAGG
C4B	Forward	GCTGAGGGGAAGTGCCCTCG
	Reverse	TGCAGGACTTGGGTGATCTTGG
SFTPC	Forward	AGCAAAGAGGTCCTGATGGAGAG
	Reverse	ACCACCACCACAACCACGAT
RET	Forward	GCATGTCAGACCCGAACTGG
	Reverse	CGCTGAGGGTGAAACCATCC
GAPDH	Forward	TCACCACCATGGAGAAGGC
	Reverse	GCTAAGCAGTTGGTGGTGCA
β-ACTIN	Forward	CATGTACGTTGCTATCCAGGC
	Reverse	CTCCTTAATGTCACGCACGAT

### Identification of DEGs and Function Annotation

For comparison of gene expression in the hippocampus of 3-, 24-, and 29 month-old mice, the following methods were used. Raw RNA-Seq reads of the gene expression series GSE61915 were downloaded from the NCBI Gene Expression Omnibus database (Stilling et al., 2014). Low quality bases and 5′ and 3′ (Q20 ≤ 20) ends of each read were trimmed using a locally developed Perl script, and adapters were removed by trimmomatic (version 0.36) (Bolger et al., 2014). The clear reads were mapped against mouse genes (GRCm38.p5) using HISAT2 (version 2.0.5) (Kim et al., 2015). The read counts were generated by BEDTools (Quinlan, 2014) and expression values were calculated using the RPKM (Reads per Kilobase per Million mapped reads) method. DEGs were obtained using the fold change of ≥1.50 or ≤0.67, and a *t*-test *p*-value of ≤0.05 was also required.

For comparison of gene expression in the hippocampus of mice treated with PBS or human cord plasma protein, human young plasma, and human advanced-age plasma, the processed data from GSE75416 were used directly (Castellano et al., 2017). DEGs were obtained using the fold change of ≥1.2 or ≤0.83, as well as a *t*-test *p*-value of ≤0.05.

Enriched functions of significantly changed genes were generated using DAVID 6.8 (Huang da et al., 2009).

### Molecular Interaction Network Construction

Human and mouse molecular interactions were collected from the BioGRID (version 3.4.153) (Chatr-Aryamontri et al., 2017) and IntAct (release 2017/9/2) (Orchard et al., 2014) databases, respectively. The BioGRID and IntAct databases contain various experimentally validated interactions, such as: two-hybrid, affinity capture, co-localization, far-western blotting, proximity label-MS, phage display, coimmunoprecipitation, pull-down, x-ray crystallography, and cross-linking. We were especially interested in the genes that directly interacted with the DEGs of special functions. All the interactions were collected from published literature, and could be retrieved from the original paper via the PubMed IDs. Thus, these data are more reliable than other computationally predicted interactions. All interactions were represented by gene symbol pairs, the BioGRID and IntAct dataset were merged based on these gene symbol pairs. Only human–human or mouse–mouse interactions were retained. For molecular interaction network construction, the first neighbors (most of which represents more reliable and usually direct interactions) of the genes related to the immune response (**Figure 2A**) were selected, and the interactions were displayed using Cytoscape 3.5.1 (Shannon et al., 2003).

The gene functions in hippocampal development were collected from Uniprot (Boutet et al., 2016) according to their GO annotation. The hippocampal development (Supplementary Table S1)-associated network was constructed using the same method. A slight difference was that all genes that interacted with hippocampal development-associated genes were DEGs, while the immune response-related genes were DEGs in the above network.

The human and mouse networks were constructed.

### Plasmid Construction

PsiRNA-YWHAE was constructed for the intracellular silence of *YWHAE* expression. The hairpin sequence containing *YWHAE* (*YWHAE*-for 5′- TCC CAG CTA ACA CTG GCG AGT CCA AGG TTT CAA GAG AAA ACC TTG GAC TCG CCA GTG TTA GCT T -3′ and *YWHAE*-rev 5′- CAA AAA GCT AAC ACT GGC GAG TCC AAG GTT TTC TCT TGA AAC CTT GGA CTC GCC AGT GTT AGC T -3′) was inserted into the psiRNA-hH1neo vector at two restriction enzyme sites (Bpi I). The negative control (psiRNA-control) with a random sequence (non-targeting siRNA) was also constructed in the same way (**Supplementary Figure S1**).

### Cell Culture and Transfection

HEK293T, an inbreeding of HEK293 (Human embryonic kidney cells 293) cells, were cultured in DMEM (Invitrogen, United States) containing 10% fetal bovine serum (Invitrogen, United States), at 37°C with 5% CO_2_. Cells were plated on 6-well plates at a density of 5 × 10^5^/ml, and the following day transfected with 4 μg psiRNA-YWHAE or psiRNA-control as a negative control and cultured for 36 h. Transfection was carried out using Lipofectamine2000 (Invitrogen, United States), according to the manufacturer’s protocol.

### Immunofluorescence

The human brain sections (provided by Huashan Hospital, Fudan University, Shanghai, China) were washed three times with PBS, treated with 0.2% Triton X-100 (diluted with PBS) for 30 min, and then blocked in 5% albumin from bovine serum for 60 min. Next, the sections were incubated with an YWHAE antibody at room temperature overnight. The following day, the sections were washed three times with PBS (5 min per wash) and then incubated with a secondary antibody for 1.5 h. Finally, the sections were stained with 4′,6-diamidino-2-phenylindole for 20 min. The expression of YWHAE was detected by confocal microscopy. The experiment protocols were approved by the Human Ethic Committee of Huashan Hospital (Fudan University, Shanghai, China), and Shanghai University Ethics Committee.

## Results

### DEGs in the Hippocampus between Young and Advanced-Age Mice

**FIGURE 1:**
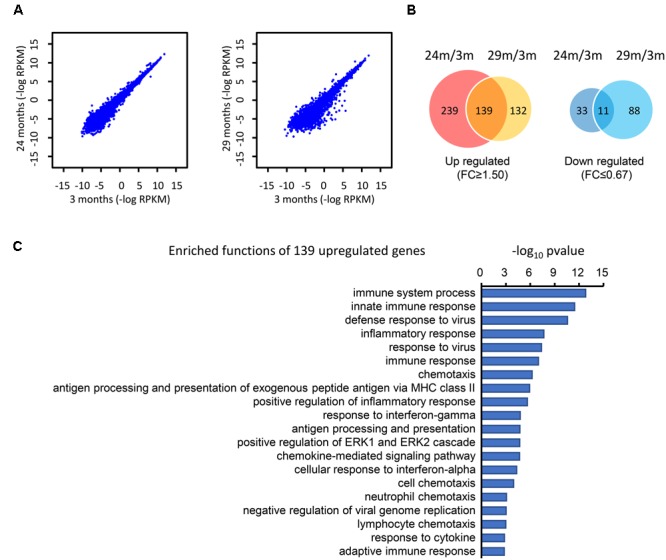
Identification of the DEGs in the hippocampus of advanced-age mice (comparison of 3-, 24-, and 29-month-old mice). **(A)** The distribution of genes in 3 month- (3M) vs. 24 month- (24M, left panel) and 3M- vs. 29 month- (29M, right panel) old mice. Each dot in the diagrams corresponds to one gene. Compared with the left panel (24 month vs. 3 month), the right panel (29 month vs. 3 month) shows a greater degree of dispersion. **(B)** Venn diagrams show the overlap of up-regulated and down-regulated genes in the 24M/3M and 29M/3M groups, respectively. **(C)** The gene function enrichment analysis of 139 up-regulated genes using the DAVID platform.

To determine the difference at the molecular level during the hippocampal aging process, we compared the gene expression in the hippocampus between young (3 months) and advanced-age (24 and 29 months) mice (Stilling et al., 2014). In comparison with 3 month-age mice, 29 month-age mice showed a larger difference than that of 24 month-age mice, as can be seen from the greater discrete degree in the scatter plot (**Figure 1A**). These DEGs were subsequently classified into two categories, which included 510 up-regulated and 132 down-regulated genes (**Figure 1B**). Specifically, 378 and 271 genes were up-regulated by at least 1.5-fold (*t*-test *p*-value ≤ 0.05) in the 24- and 29 month-age mice respectively and 139 genes were consistently up-regulated (**Figure 1B**). 44 and 99 genes, respectively, were down-regulated by at least 1.5-fold, and 11 genes were collectively down-regulated (**Figure 1B**). The small overlap between the up and down regulated genes of the 24 and 29 months old mice is due to the variable gene expressions between these stages, indicating the aging is a dynamically changing process. It can be inferred that there are more genes up-regulated than those down-regulated. Moreover, the number of overlapping genes in the up-regulated group (139) is substantially larger than in the down-regulated group (11), illustrating that these common genes may play a general role during the process of hippocampal aging. Furthermore, DAVID (Huang da et al., 2009) functional annotation shows that the majority of the up-regulated genes are associated with the innate and inflammatory immune responses (**Figure 1C**). Interestingly, the immune response-related functions were also found to have significant changes during aging (High, 2004; Weinberger et al., 2008; Balivada et al., 2017), indicating that immune response-related genes may play important roles during the hippocampal aging process.

### Immune Response-Associated Molecular Interaction Network during Hippocampal Aging

**FIGURE 2:**
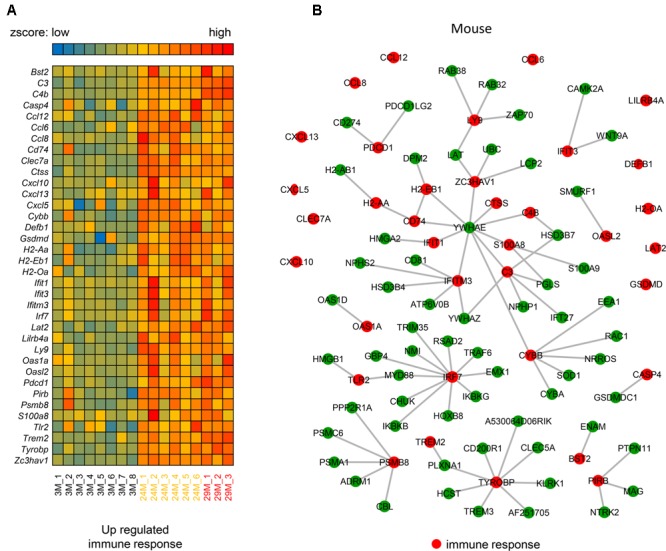
The molecular interaction network of up-regulated genes that are associated with the immune response in mice. **(A)** Heatmap of up-regulated immune response-related genes in the 3 age groups of mice (3M, *n* = 8; 24M, *n* = 6; 29M, *n* = 3). Red represents genes with high expression and blue represents those with low expression. **(B)** The molecular interaction network related to the immune response (red nodes) and their partner genes (green nodes; 61). A gray line represents an interaction between two nodes.

The above analysis demonstrates the importance of the immune response (**Figure 1C**) in hippocampal aging, however, the underlying mechanism of this genes network is still unknown. Thus, we investigated the molecular interactions (see section “Materials and Methods”) of these genes in the BioGRID (Chatr-Aryamontri et al., 2017) and IntAct (Orchard et al., 2014) databases. The molecular interactions formed a network (**Figures 2A,B**) that included 97 nodes and 78 edges, with 24 immune response-related genes being up-regulated (indicated in red nodes) and interacted with its partners. Meanwhile, there are 12 single genes without interactions to others. Through interaction with these 24 genes, their partner genes (green nodes) may indirectly participate in the aging-related immune response. Notably, *Ywhae* is shown to be linked to many immune response-related genes (**Table 2**), forming a hub in the network (**Figure 2B**). Considering its connection to many immune response genes (**Figure 2B**), it can be speculated that *Ywhae* may not only have an influence in the nervous system (Baxter et al., 2002; Toyo-oka et al., 2003) but also in the immune response during the aging process.

**Table 2:** The up-regulated immune response-related genes that interacted with *Ywhae*.

Index	Gene symbol	Gene name
1	*Cybb*	Cytochrome b-245, beta polypeptide
2	*Ifit1*	Interferon-induced protein with tetratricopeptide repeats 1
3	*Ctss*	Cathepsin S
4	*Ifitm3*	Interferon induced transmembrane protein 3
5	*H2-Eb1*	Histocompatibility 2, class II antigen E beta
6	*Cd74*	CD74 antigen
7	*C4b*	Complement component 4B
8	*C3*	Complement component 3
9	*S100a8*	S100 calcium binding protein A8
10	*Zc3hav1*	Zinc finger CCCH type, antiviral 1

We also investigated the interactions in human. The hub gene *Ywhae* of the above immune response associated molecular interaction network in mice was not observed in human (**Supplementary Figure S2**). However, this does not mean the immune response associated molecular interaction network is not conserved between human and mouse, since the interaction dataset of both human and mouse were incomplete, the overlapped interactions were limited.

### Membrane Receptors Are Down-Regulated during the Hippocampal Aging Process

**FIGURE 3:**
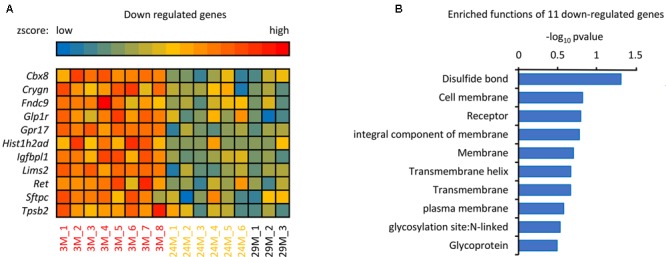
The analysis of down-regulated genes in the hippocampus of advanced-age mice. **(A)** Heatmap of the 11 down-regulated genes in mice of different ages. The red and blue color spectrum represents high and low expressed genes, respectively (3M, *n* = 8; 24M, *n* = 6; 29M, *n* = 3). **(B)** Function enrichment analysis of 11 down-regulated genes. The DAVID platform was used for analysis; the 11 down-regulated genes have 10 functions exhibited in the column diagram. The majority of the genes were enriched in disulfide bonds.

In addition to the 139 up-regulated genes (**Figure 1B**), 11 down-regulated genes were also observed, as shown in **Figure 3**. The majority of these genes, including *Ret, Sftpc, Gpr17, Tpsb2*, and *Glp1r* are associated with disulfide bonds that are crucial to protein structure (Butera et al., 2014), it has been reported that disulfide bonds may influence the function of proteins in the blood (Butera et al., 2014). In addition to disulfide bonds, cell membrane and receptor-associated genes were also observed, including *Ret, Lims2, Gpr17* and *Glp1r*. Membrane receptors embedded in the plasma membrane bind extracellular molecules and allow communication between the extracellular and intracellular space, meaning that environmental exposure may influence aging through transmembrane receptors (Bettio et al., 2017). As shown in **Figure 3**, *Sftpc*, for example, encodes the membrane protein surfactant-associated protein C (SP-C) (Glasser et al., 2013). It has been reported that *Sftpc* plays an important role in innate host defense, enhancing macrophage-mediated phagocytosis and clearing, and limiting inflammatory responses (Glasser et al., 2008), which are all important during aging (**Figure 1C**). Further, GPR17 is a marker for progenitor progression within the oligodendroglia lineage (Boda et al., 2011) and is expressed in all regions of the brain, indicating that downregulation of GPR17 in our network may interfere with normal brain function and lead to aging. In conclusion, these down-regulated genes take part in many different regulatory pathways together with the up-regulated genes. The current results demonstrate that the identified genes have important biological significance in hippocampal aging research.

### DEGs during Hippocampal Aging Are Closely Related to Hippocampal Development

**FIGURE 4:**
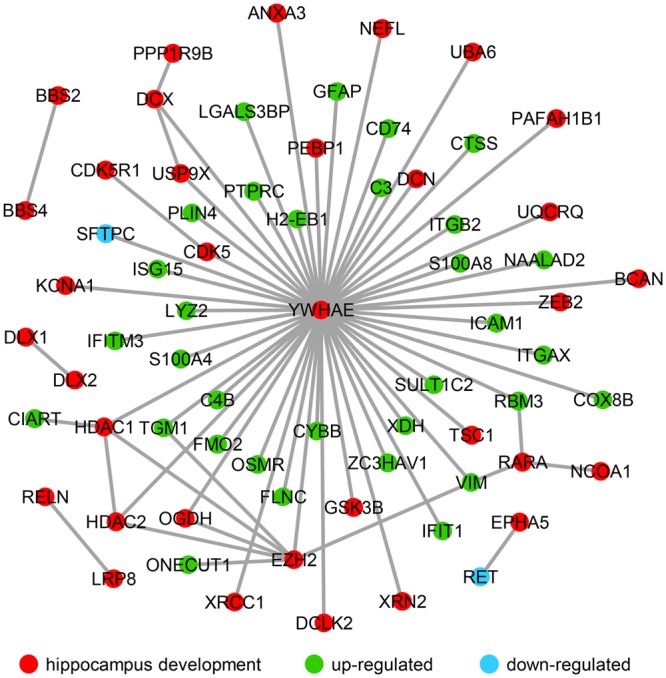
The molecular interaction network revealed the up- and down-regulated genes that are strongly associated with hippocampal development. Hippocampal development genes (34) are marked with red nodes, and the 32 green nodes refer to the up-regulated genes in advanced-age compared with young mice. The down-regulated genes (*Sftpc* and *Ret*) are labeled with blue nodes.

It has been reported that a change in hippocampal volume and structure is observed with age-related decline in learning and memory (Verbitsky et al., 2004; Arias-Cavieres et al., 2017; Bettio et al., 2017; Moret-Tatay et al., 2017). Additionally, in the aging hippocampus, neurobiological alterations have been clearly observed including those that alter intracellular signaling and gene expression, and cause neuroinflammation response (Bettio et al., 2017). In recent years, studies regarding of the age-related hippocampus have focused on cognitive decline and impaired memory (Arias-Cavieres et al., 2017; Bettio et al., 2017; Moret-Tatay et al., 2017). Several studies revealed the DEGs in hippocampus between young and the aging mice linked to the cognitive deficient (Xiong et al., 2000; Cheng et al., 2007; Thomas et al., 2014), synaptic plasticity (Dempsey and Ali, 2014) and various dysregulated pathways related to immune and inflammatory response (Landel et al., 2016). In order to understand how significantly up- and down-regulated genes are involved in hippocampal function during aging, their interactions were investigated using public databases and established a network using the privous methods (von Mering et al., 2003; Liu and Zhao, 2004). Interestingly, most of these genes interact with *Ywhae* (**Figure 4**), which is the hub gene in the immune response network during hippocampal aging (**Figure 2B**). Nucleotide polymorphisms in *Ywhae* would lead to abnormal hippocampal development (Kido et al., 2014), implying that *Ywhae* maybe influence the function of hippocampus during aging through certain signaling pathway. As shown in **Figure 4**, YWHAE interacts with VIM, which is an integral regulator of cell adhesion (Dave and Bayless, 2014), neurite extension (Dave and Bayless, 2014), and myelination (Triolo et al., 2012), suggesting that *Ywhae* may be involved in the regulation of neural function through *Vim* during aging. Furthermore, GFAP, which is expressed in astrocytes of the central nervous system (Eng and Ghirnikar, 1994), is the partner of YWHAE (**Figure 4**) and is important for the brain to accommodate neural activities or changes during development (Kim et al., 2011). Moreover, GFAP also plays a crucial role in neuro-inflammation of central nervous system injury (Otani et al., 2003), which is consistent with the speculation that *Ywahe* is involved in the immune response (**Figure 2B**) together with GFAP. In conclusion, the interaction between YWHAE and its partners is obviously important for hippocampal development in the nervous system, implying an important function of these genes in the aging process. Interestingly, YWHAE interacts with C4B, which is up-regulated in aging mice and may influence the autoimmune process in diseases (Paul et al., 2002) or modulate interstitial inflammation (Welch et al., 2001), participating in the immune response. Moreover, C4B is also associated with ITGB2, which regulates the lineage distribution of hematopoietic cells in the blood and bone marrow (Gomez and Doerschuk, 2010), implying that *Ywhae* may be involved in different signaling pathways contributing to the aging process. Essentially, aging is a slow and complicated process that is accompanied by numerous changes in gene expression, especially those related to hippocampal development. This also partially explains why these genes influence hippocampal development and impair memory and learning ability, accelerating aging.

### Verification of Gene Expression in the Interaction Network by QPCR

**FIGURE 5:**
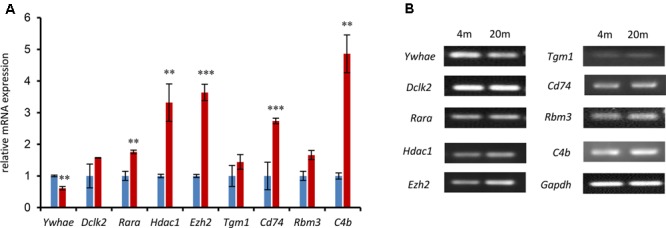
The expression of nine randomly selected genes was identified by qPCR in the 4- and 20-month-old hippocampus. **(A)** The relative genes were randomly selected from the network in **Figure 4** and confirmed by qPCR. The blue columns refer to mRNA levels of these genes in 4-month-old mice, and the red columns represent those in the 20-month-old mice. The 4M mice are regarded as a control (^∗∗∗^*p* ≤ 0.001; ^∗∗^*p* ≤ 0.05; two-sided *t*-test; *n* ≥ 3). **(B)** QPCR products were mixed with DNA loading buffer and separated on a 1% agarose gel, detected using ethidium bromide staining, and visualized on a gel imaging system. GAPDH was used as control in each mRNA transcript. Data are represented as the mean ± SEM, *n* ≥ 3. ^∗^*p* ≤ 0.05; ^∗∗^*p* ≤ 0.01; ^∗∗∗^*p* ≤ 0.001.

In order to further verify the expression of the genes in our current research (**Figure 4**), a number of genes in the network were randomly chosen and their expression was evaluated by qPCR in advanced-age and young mice. Hippocampal tissues were extracted from 4- and 20 month-old male mice and the mRNA amplified and compared with that of 4 month-old mice as a control. As shown in **Figure 5**, the majority of hippocampal development genes (*Dclk2, Rara, Hdac1*, and *Ezh2*) were up-regulated, with the exception of *Ywhae*. Moreover, *Tgm1, Cd74, Rbm3*, and *C4b* were also up-regulated compared with the young mice group, which is consistent with the network shown in **Figure 4**.

### Interactions between YWHAE and Its Partners Are Conserved between Humans and Mice

**FIGURE 6:**
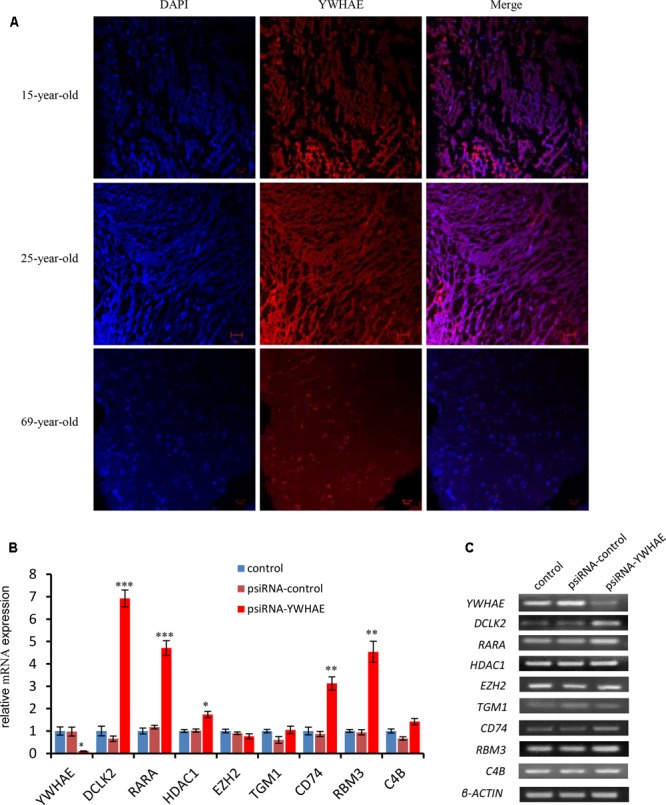
The expression of *YWHAE* and its partner genes in the human brain and HEK293T cells. **(A)** Immunofluorescence staining showing the low expression of YWHAE in the aged human brain compared with young human brain (red:YWHAE, blue:nuclei). **(B)** QPCR of gene expression in HEK293T cells after silencing of the expression of *YWH*AE. The eight genes were assessed by qPCR in the control group, vehicle group (transfected with psiRNA-control), and silencing group (transfected with psiRNA-YWHAE), in HEK293T cells. **(C)** QPCR products were mixed with DNA loading buffer and separated on 2% agarose gels, detected using ethidium bromide staining, and visualized on a gel imaging system. β-actin was used as a loading control for each mRNA transcript. Data are represented as the mean ± SEM, *n* ≥ 3. ^∗^*p* ≤ 0.05; ^∗∗^*p* ≤ 0.01; ^∗∗∗^*p* ≤ 0.001.

In order to further explore the expression pattern of YWHAE (**Figure 4**) in humans, the expression of YWHAE was evaluated in human brain (**Figure 6A**). It was found that compared with the young human brain (15- and 25-years-old), YWHAE is down-regulated in the older human brain (69-year-old). These results are consistent with those seen in mice (**Figures 5A,B**). Furthermore, to evaluate whether the interaction network of YWHAE in mice is conserved in humans, qPCR was carried out to detect the mRNA expression in HEK293T cells after silencing *YWHAE*. The psiRNA-YWHAE vector was constructed to silence it in HEK293T cells. As shown in **Figures 6B,C**, with the obvious down-regulated expression of YWHAE, the mRNA levels of DCLK2, RARA, HDAC1, CD74, and RBM3 were significantly increased in HEK293T cells, which is consistent with the results observed in mice (**Figure 5A**). However, due to the diversity of gene expression in different species, not all genes exhibit the same expression pattern (**Figure 5A**). For instance, there is no significant difference in the expression of EZH2, TGM1, or C4B at the mRNA level. In conclusion, the gene expression pattern in humans is consistent with that in mice, indicating that the interaction network of YWHAE is conserved between humans and mice.

### The Genes That Contribute to the Revitalization of Advanced-Age Mice Are Different from the Genes Which Are Induced by Aging

**FIGURE 7:**
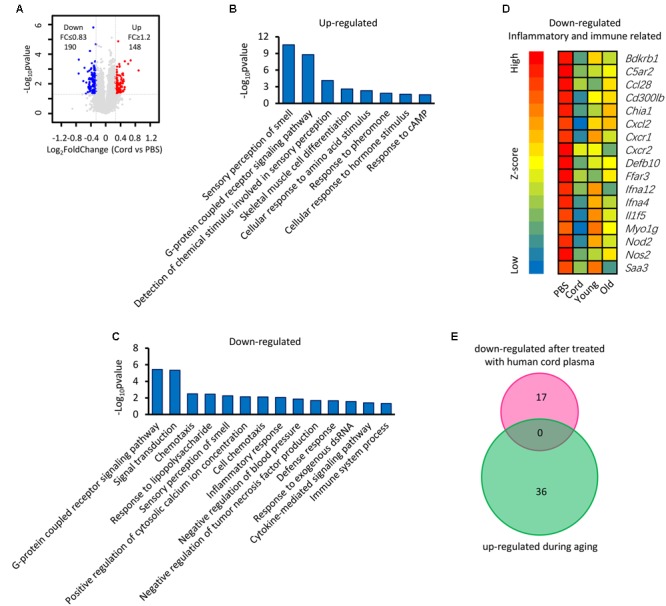
Identification of genes involved in the revitalization of advanced-age mice compared with young and advanced-age mice. **(A)** Scatter plot of gene expression following treatment with umbilical cord blood protein or PBS. Each point refers to one gene. **(B,C)** The DAVID platform was used to analyze the function of DEGs. **(D)** Heatmap of down-regulated genes related to inflammatory and immune responses. The first group is treated with PBS and regarded as a control, the second is treated with human umbilical cord protein, and the remaining two groups are the young and the advanced-age group, respectively. **(E)** Venn diagrams show the overlap of inflammatory and immune response-related genes that up-regulated during aging and those down-regulated following treatment with human cord plasma.

It has been reported that human umbilical cord plasma proteins can revitalize hippocampal function in advanced-age mice (Castellano et al., 2017), and that tissue inhibitor of metalloproteinases 2, which is an umbilical cord plasma protein, provides a reservoir for the neuroplasticity-promoting process. In order to gain more information regarding this process, microarray data from mice treated with human cord plasma (Castellano et al., 2017) were re-analyzed (**Figure 7**). In comparison with the PBS control, a total of 148 up-regulated and 190 down-regulated genes (up- or down-regulated by least 1.2-fold, *p*-value ≤ 0.05) were observed in the samples treated with human cord plasma (**Figure 7A**). Function enrichment analysis indicates that these genes exhibit functions including sensory perception, skeletal muscle cell differentiation, G protein-coupled receptor signaling, and detection of chemical, amino acid, hormone stimuli (**Figures 7B,C**). The hypothesis exists that changes in textural perception (Conroy et al., 2017) and loss of skeletal muscle stem cells with age may drive the degeneration of age-related neuromuscular junctions (Liu et al., 2017), indicating that genes with these functions (**Figures 7B,C**) may participate in aging progression or the revitalization progress. Interestingly, certain inflammatory- and immune response-related genes were also observed among those genes that were down-regulated (**Figure 7D**). However, no overlapping genes (**Figure 7E**) were found between those up-regulated during hippocampal aging (**Figure 2A**) and those down-regulated following treatment with human cord plasma (**Figure 7D**), indicating that mice adopt a different process to become younger after human umbilical cord plasma treatment. Our current data suggest that advanced-age mice do indeed benefit from these DEGs; however, it is noteworthy that this appears not to be a reverse process from advanced-age to young mice following treatment with umbilical cord plasma. In other words, the genes which contribute to the revitalization of advanced-age mice are different from the genes which are induced by aging during the normal hippocampal aging process.

## Discussion

In the present study, we identified DEGs between young and advanced-age mice, and found that the majority of the up-regulated genes in DEGs were enriched in the immune response process (**Figure 1**). Moreover, membrane receptor-associated genes were down-regulated (**Figure 3**). The analysis of the molecular interaction network indicated that *Ywhae* plays important roles during hippocampal aging (**Figure 2B**). Subsequently, we found that the interactions between YWHAE and its partners are conserved between humans and mice (**Figures 5, 6**). Furthermore, our results also showed that the genes that contribute to the revitalization of advanced-age mice are different from the genes induced by aging (**Figure 7**). Our data assigned novel roles for genes during hippocampal aging progression and provided further theoretical evidence for future studies exploring the underlying mechanisms of hippocampal aging and anti-aging-related disease therapy.

The progression of aging is complex. In order to explore the mystery of aging, many researchers have used microarray-related approaches to perform aging-related research (Bishop et al., 2010; Stilling et al., 2014). In addition, transcriptional profiles were focused on (Pawlowski et al., 2009; Bishop et al., 2010; Stilling et al., 2014) with different research purposes. In our current research, this common approach was also used to analyze DEGs associated with hippocampal aging progression. Some researchers have revealed that DEGs are closely linked to synaptic plasticity (Dempsey and Ali, 2014), cognitive impairment (Cheng et al., 2007; Park et al., 2015), age-associated spatial learning impairment (ASLI) (Uddin and Singh, 2013), degenerative diseases (Xiong et al., 2000; Kikuchi et al., 2013) and even various dysregulated pathways (Landel et al., 2016). In addition, it has been reported that the immune system is dysregulated in the aging brain (Patterson, 2015), which was consistent with our results that the majority of up-regulated genes were enriched in the immune response-related process (**Figure 1C**).

In order to build a reliable network, we collected interaction data from the BioGRID (Chatr-Aryamontri et al., 2017) and IntAct (Orchard et al., 2014) databases. These databases contain various experimentally validated interactions curated from published literature. Moreover, the networks we built were first-neighbor networks, since the first neighbors represent the most reliable interactions. The second-neighbor network may contain many genes and interactions that are directly linked to the first neighbors but not the genes that we are interested in (immune response or hippocampal development), thus, the center of the network may be shifted. However, we also constructed a second-neighbor network for mice based on the immune response-related genes (**Supplementary Figure S3**). We found that the network is huge, containing 5,283 genes and 19,641 interactions. Link degree analysis of each node in the network indicated that *Ywhae, Eed, Ywhaz, Ubc, Hsd3b4, Pgls, Hsd3b7, Ywhab, Hmga2*, and *Dlg4* were the top 10 genes with the most interactions with other genes. YWHAE still had the most links with other genes, which is the similar phenomenon as seen in **Figure 2B**. Most interestingly, YWHAE, YWHAZ, and YWHAB belong to the 14-3-3 protein family, which mediate signal transduction by binding to phosphoserine-containing proteins (Conklin et al., 1995). These results implied that the 14-3-3 protein family has a potential role during the hippocampal aging process. We also checked the conversation of the hippocampus development associated network in human and mice (**Supplementary Figure S4** and **Figure 4**). In mice, *Ywhae* was linked to many up-regulated DEGs (**Figure 4**, green nodes), indicating *Ywhae* may be involved in the regulation of those genes (green nodes) during hippocampus development. However, the same situation was not observed in human (**Supplementary Figure S4**). Considering the incompletion of the interaction data deposited in both BioGRID and IntAct, this may be due to the largely un-delineated overlapping of the two datasets.

Consistent with the previous studies (Stilling et al., 2014), the constructed network implied that the up-regulated DEGs between young and advanced-age mice (**Figure 2A**) interact with their partners to participate in the immune response and facilitate hippocampal aging progression (**Figure 2B**). As people become older, their immune systems become weaker and antibody responses are slow (Montecino-Rodriguez et al., 2013), which induces many dysfunctions (Weigle, 1989). This could explain why older people are more likely to get sick. However, due to the limitations of the database, there were 12 genes (Supplementary Table S2) that did not appear in this network, meaning that these genes showed no functional associations with the other genes. Nevertheless, this does not mean that these genes are not related to hippocampal aging. For instance, CXCL5, a CXC-type chemokine, has been reported to be secreted by aging prostate stroma, implying its important function in aging progression (Begley et al., 2008). As shown in **Figure 3**, five down-regulated genes are associated with disulfide bonds, and oxidation of protein sulfhydryl groups to disulfide groups occurs as a normal part of human aging (Takemoto, 1996), indicating that disulfide bonds play an important unclarified role in aging, and further research is needed to explore the underlying mechanisms. In addition, cell membrane and receptor were also enriched in **Figure 3B**. For instance, *Ret* is a crucial gene that plays an important role in the development and function of the nervous system (Gabreski et al., 2016), as well as driving HSCs survival. Ablation of *Ret* cannot only impair the number of HSCs but also influences its normal differentiation potential (Fonseca-Pereira et al., 2014). The down-regulation of *Ret* (**Figure 3A**) implies that HSC function is impaired during aging. Most importantly, the enrichment of many down-regulated membrane proteins may also indicate weaker perception ability to environmental exposure of older people, since many membrane proteins participate in different signaling pathways that transduce extracellular information across the plasma membrane. In conclusion, this indicates that these genes, although enriched in different functions, may work together to promote hippocampal aging through regulating cell communication.

Our results also showed that no overlapping genes (**Figure 7E**) were found between those up-regulated during hippocampal aging (**Figure 2A**) and those down-regulated following treatment with human cord plasma (**Figure 7D**), indicating that the revitalization induced by the treatment of human umbilical cord blood plasma protein is not a simple reverse process of natural aging progression. We noticed that the mice strains from the other research studies are different. Immuno-deficient (NSG) mice were chosen for the injection experiment, since the mice can receive intravenous injection of human plasma without an adverse immune response (Castellano et al., 2017), while, C57BL/6J mice were used for detecting gene expression differences in the other research. This likely provides an explanation for the lack of overlap in gene expression. Another possibility for our results could be that the mice treated with PBS and human cord plasma was 14-months-old rather than 24- or 29-months-old. The distance in age of the mice may have led to differences in gene expression.

## Conclusion

The present study provides a more comprehensive analysis of aging and anti-aging treatment using a biological systems approach. Our data suggested that a large number of immune and inflammatory response-related genes were up-regulated in the aged hippocampus, and that membrane receptor-associated genes were down-regulated. Moreover, *Ywhae* in the predicted network may be assigned novel roles during hippocampal aging progression. The results also implied that the revitalization of advanced-age mice may be not a simple reverse process of normal aging progression. Considering the association of aging-related diseases such as Alzheimer’s disease, hypertension, and cancer, our study provides further theoretic evidence for future studies exploring the underlying mechanisms of aging and anti-aging-related disease therapy.

## Author Contributions

JW and TW contributed to the design, analysis, and interpretation of data for the study. JW and QL drafted the work, interpreted the data and conducted the experiments. QL, FZ, JL, WL, and KW conducted the experiments. YK, TW, and YG collected data. JX and TQW finally approved the version to be published.

## Conflict of Interest Statement

The authors declare that the research was conducted in the absence of any commercial or financial relationships that could be construed as a potential conflict of interest.

## Supplementary Material

FIGURE S1 The interfere fragment of *YWHAE* was successfully inserted into the vector of psiRNA-hH1neo. **(A)** The diagram of plasmids construction. The sequence of YWHAE was marked in yellow. **(B)** The plasmids of psiRNA-hH1neo and psiRNA-YWHAE were identified by 0.6% agarose gels. The arrow shows the recombinant plasmid. **(C)** The product of plasmids that were cut by single enzyme (Xba I) was identified by 0.6% agarose gels. The arrows shows the sequence was successfully inserted into psiRNA-hH1neo.

FIGURE S2 The molecular interaction network related to the immune response in human. Red nodes represent the immune responds genes. A gray line represents an interaction between two nodes.

FIGURE S3 The second neighbor molecular interaction network related to the immune response in mice.

FIGURE S4 The human molecular interaction network involved in hippocampus development. Hippocampus development associate genes (37) are marked with red nodes. The 13 green nodes refer to the homologous DEGs in the mice.

## Abbreviations

BioGRID: The Biological General Repository for Interaction Datasets
DAVID: The Database for Annotation, Visualization and Integrated Discovery
DEG: differentially-expressed gene
GFAP: glial fibrillary acidic protein
HSCs: hematopoietic stem cells
PBS: phosphate-buffered saline
qPCR: real-time quantitative polymerase chain reaction
