# Supplementary figures and images for: Biosystems Study of the Molecular Networks Underlying Hippocampal Aging Progression and Anti-aging Treatment in Mice
Source: Front Aging Neurosci. 2017 Dec 6;9:393. doi: 10.3389/fnagi.2017.00393 (PMC5735351; doi:10.3389/fnagi.2017.00393)

A

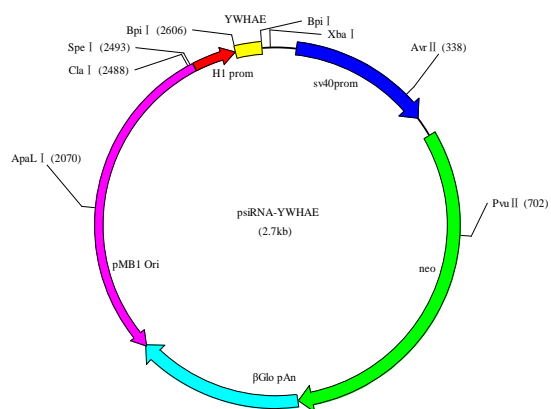

B

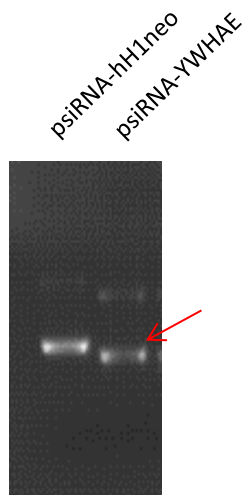

C

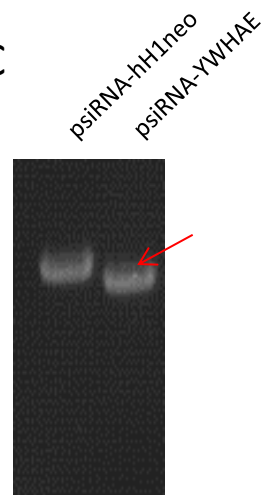

Supplement: FIGURE S1 — The interfere fragment of YWHAE was successfully inserted into the vector of psiRNA-hH1neo. (A) The diagram of plasmids construction. The sequence of YWHAE was marked in yellow. (B) The plasmids of psiRNA-hH1neo and psiRNA-YWHAE were identified by 0.6% agarose gels. The arrow shows the recombinant plasmid. (C) The product of plasmids that were cut by single enzyme (Xba I) was identified by 0.6% agarose gels. The arrows shows the sequence was successfully inserted into psiRNA-hH1neo. [file Image_1.pdf]

Human

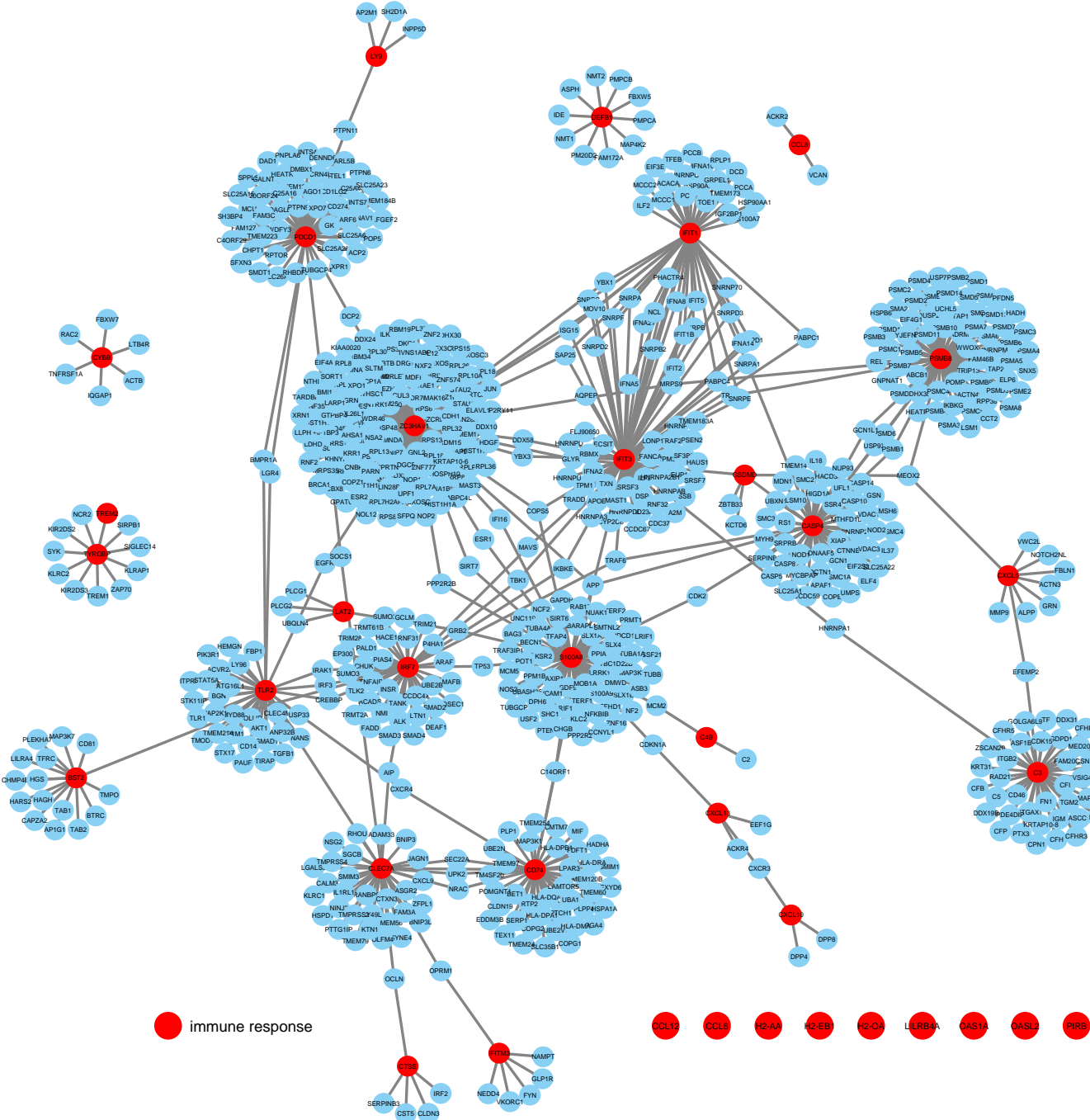

Supplement: FIGURE S2 — The molecular interaction network related to the immune response in human. Red nodes represent the immune responds genes. A gray line represents an interaction between two nodes. [file Image_2.pdf]

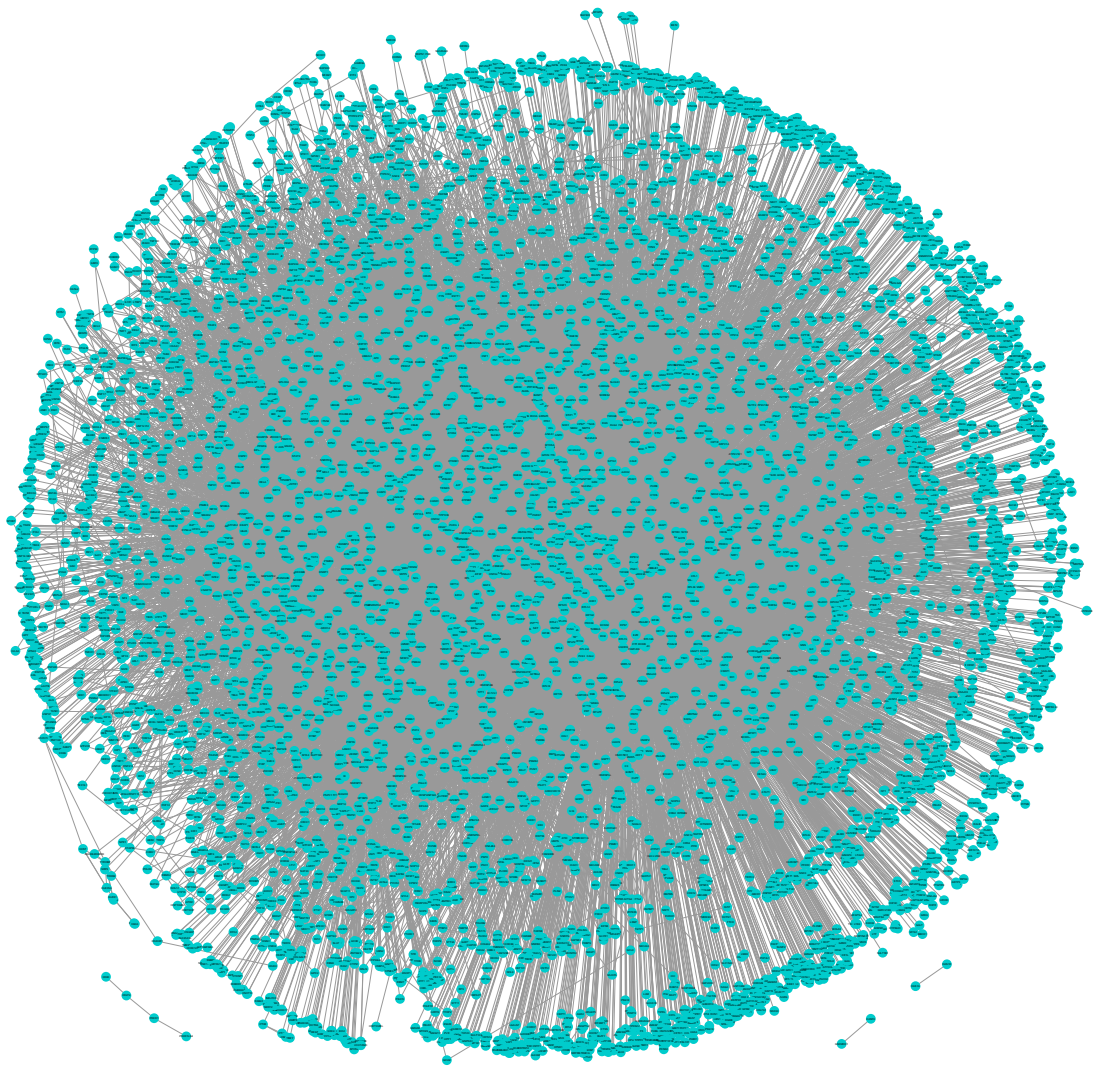

Supplement: FIGURE S3 — The second neighbor molecular interaction network related to the immune response in mice. [file Image_3.pdf]

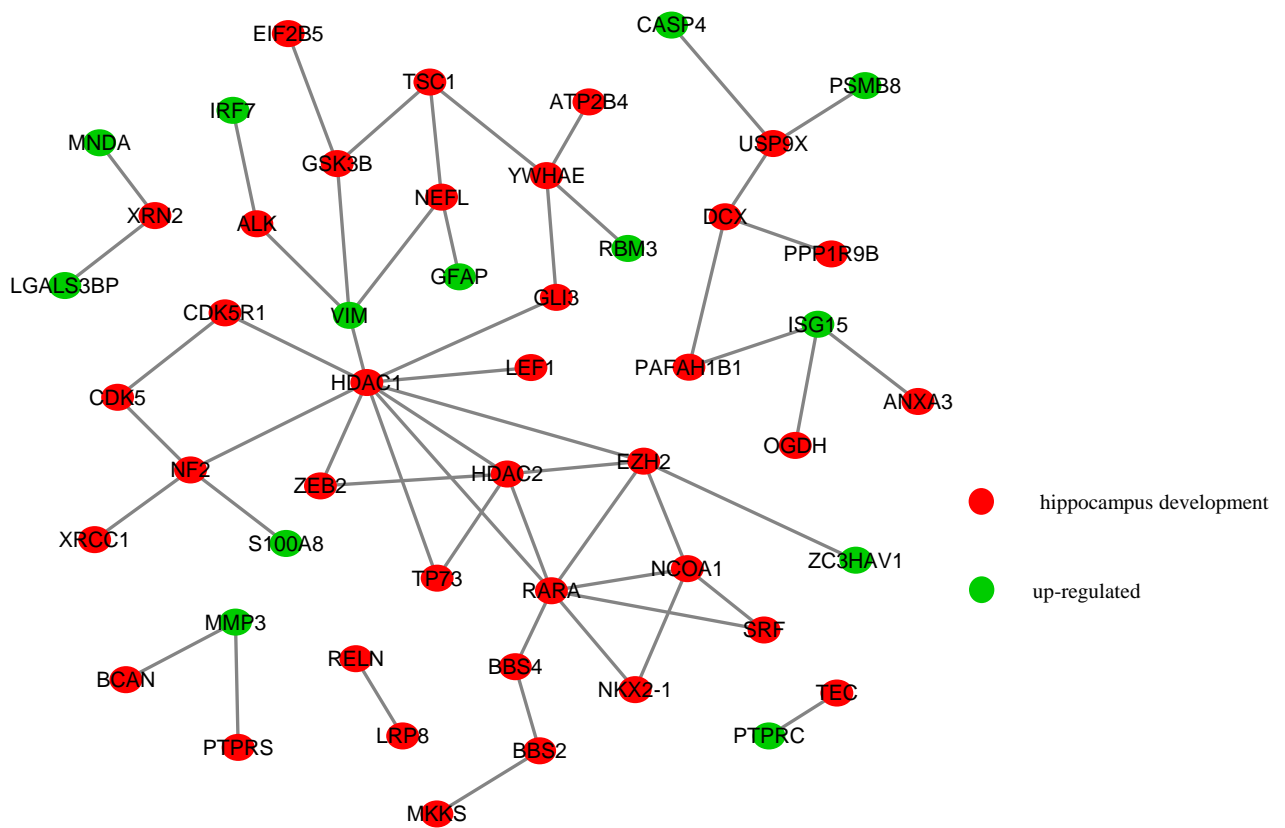

Supplement: FIGURE S4 — The human molecular interaction network involved in hippocampus development. Hippocampus development associate genes (37) are marked with red nodes. The 13 green nodes refer to the homologous DEGs in the mice. [file Image_4.pdf]
