# Supplementary material for: Biosystems Study of the Molecular Networks Underlying Hippocampal Aging Progression and Anti-aging Treatment in Mice
Source: Front Aging Neurosci. 2017 Dec 6;9:393. doi: 10.3389/fnagi.2017.00393 (PMC5735351; doi:10.3389/fnagi.2017.00393)
Supplement: Supplementary file 5 [file Table_1.pdf]

**TABLE S1. The genes that related to hippocampus development**

| Index | Gene Symbol | Index | Gene Symbol | Index | Gene Symbol | Index | Gene Symbol |
|---|---|---|---|---|---|---|---|
| 1 | 5ART2 | 34 | FAM | 67 | MFSD2 | 100 | SIP1 |
| 2 | 5HT5A | 35 | FGF13 | 68 | MFSD2A | 101 | SLC32A1 |
| 3 | ALK | 36 | FHF2 | 69 | MK | 102 | SRC1 |
| 4 | ANX3 | 37 | GLD2 | 70 | MKKS | 103 | SRD5A2 |
| 5 | ANXA3 | 38 | GLI3 | 71 | NCK5A | 104 | SRF |
| 6 | APOER2 | 39 | GPCR15 | 72 | NCK5AI | 105 | TEC |
| 7 | ATP2B4 | 40 | GSK3B | 73 | NCOA1 | 106 | TES-1 |
| 8 | BBS2 | 41 | HDAC1 | 74 | NEFL | 107 | TES1 |
| 9 | BBS4 | 42 | HDAC2 | 75 | NEPH2 | 108 | TITF1 |
| 10 | BBS6 | 43 | HTR5A | 76 | NF-2 | 109 | TP73 |
| 11 | BCAN | 44 | ID-4 | 77 | NF2 | 110 | TRP73 |
| 12 | BSK | 45 | ID4 | 78 | NF68 | 111 | TSC1 |
| 13 | CDK5 | 46 | IDB4 | 79 | NFL | 112 | TTF1 |
| 14 | CDK5R | 47 | JMJD3 | 80 | NKX-2.1 | 113 | UBA6 |
| 15 | CDK5R1 | 48 | KCNA1 | 81 | NKX2-1 | 114 | UBE1L2 |
| 16 | CDK5R2 | 49 | KDM6B | 82 | NLS1 | 115 | UQCRQ |
| 17 | CDKN5 | 50 | KIAA0243 | 83 | NR1B1 | 116 | USP9X |
| 18 | CRK6 | 51 | KIAA0346 | 84 | NR4A3 | 117 | VGAT |
| 19 | DCAMKL2 | 52 | KIAA1867 | 85 | OGDH | 118 | VIAAT |
| 20 | DCLK2 | 53 | KIAA4192 | 86 | P73 | 119 | WNT-3A |
| 21 | DCN | 54 | KIF14 | 87 | PAFAH1B1 | 120 | WNT3A |
| 22 | DCX | 55 | KIRREL3 | 88 | PAFAHA | 121 | XRCC-1 |
| 23 | DHM1 | 56 | LEF-1 | 89 | PAPD4 | 122 | XRCC1 |
| 24 | DLX1 | 57 | LEF1 | 90 | PBP | 123 | XRN2 |
| 25 | DLX2 | 58 | LHX5 | 91 | PEBP | 124 | YWHAE |
| 26 | DRD1 | 59 | LIS-1 | 92 | PEBP1 | 125 | YY1BP |
| 27 | DRD1A | 60 | LIS1 | 93 | PLXNA3 | 126 | ZBTB18 |
| 28 | EHK1 | 61 | LMX1A | 94 | PPP1R9B | 127 | ZEB2 |
| 29 | EIF2B5 | 62 | LRP8 | 95 | PTPRS | 128 | ZFHX1B |
| 30 | ENX1H | 63 | MAS | 96 | RARA | 129 | ZFP238 |
| 31 | EPHA5 | 64 | MAS-1 | 97 | RELN | 130 | ZFX1B |
| 32 | EZH2 | 65 | MAS1 | 98 | RL | 131 | ZNF238 |
| 33 | FAFL | 66 | MDK | 99 | RP58 | | |
