# Supplementary material for: Biosystems Study of the Molecular Networks Underlying Hippocampal Aging Progression and Anti-aging Treatment in Mice
Source: Front Aging Neurosci. 2017 Dec 6;9:393. doi: 10.3389/fnagi.2017.00393 (PMC5735351; doi:10.3389/fnagi.2017.00393)
Supplement: Supplementary file 6 [file Table_2.pdf]

Table S2. The twelve genes that did not appear in the up-regulated network

Index	Gene Symbol	Gene Name
1	*Ccl12*	chemokine (c-c motif) ligand 12
2	*Ccl6*	chemokine (c-c motif) ligand 6
3	*Ccl8*	chemokine (c-c motif) ligand 8
4	*Clec7a*	c-type lectin domain family 7,member a
5	*Cxcl13*	chemokine (c-x-c motif) ligand 13
6	*Cxcl5*	chemokine (c-x-c motif) ligand 5
7	*Defb1*	defensin beta 1
8	*Gsdmd*	gasdermin D
9	*H2-Oa*	histocompatibility 2, O region alpha locus
10	*Lat2*	linker for activation of T cells family, member 2
11	*Lilrb4a*	leukocyte immunoglobulin-like receptor, subfamily B, member 4A
12	*Cxcl10*	chemokine (C-X-C motif) ligand 10
